# Identifying Nonclinical Factors Associated With 30-Day Readmission in Patients with Cardiovascular Disease: Protocol for an Observational Study

**DOI:** 10.2196/resprot.7434

**Published:** 2017-06-15

**Authors:** Matthew E Dupre, Alicia Nelson, Scott M Lynch, Bradi B Granger, Hanzhang Xu, Janese M Willis, Lesley H Curtis, Eric D Peterson

**Affiliations:** ^1^ Duke Clinical Research Institute Duke University Durham, NC United States; ^2^ Department of Sociology Duke University Durham, NC United States; ^3^ Department of Community and Family Medicine Duke University Durham, NC United States; ^4^ Duke University School of Nursing Duke University Durham, NC United States

**Keywords:** cardiovascular disease, readmission, socioeconomic status, psychosocial factors, United States, eHealth, observational study

## Abstract

**Background:**

Cardiovascular disease (CVD) is the leading cause of hospitalization in older adults and high readmission rates have attracted considerable attention as actionable targets to promote efficiency in care and to reduce costs. Despite a plethora of research over the past decade, current strategies to predict readmissions have been largely ineffective and efforts to identify novel clinical predictors have been largely unsuccessful.

**Objective:**

The objective of this study is to examine a wide array of socioeconomic, psychosocial, behavioral, and clinical factors to predict risks of 30-day hospital readmission in cardiovascular patients.

**Methods:**

The study includes patients (aged 18 years and older) admitted for the treatment of cardiovascular-related illnesses at the Duke Heart Center, which is among the nation’s largest and top-ranked cardiovascular care hospitals. The study uses a novel standardized survey to ascertain data on a comprehensive array of patient characteristics that will be linked to their electronic medical records. A series of univariate and multivariate models will be used to estimate the associations between the patient-level factors and 30-day readmissions. The performance of the risk models will be examined based on 2 components of accuracy—model calibration and discrimination—to determine how closely the predicted outcome agrees with the observed (actual) outcome and how well the model distinguishes patients who were readmitted and those who were not. The purpose of this paper is to present the protocol for the implementation of this study.

**Results:**

The study was launched in February 2014 and is actively recruiting patients from the Heart Center. Approximately 550 patients have been enrolled to date and the study is expected to continue recruitment until February 2018. Preliminary results show that participants in the study were aged 63.6 years on average (SD 14.0), predominately male (61.2%), and primarily non-Hispanic white (64.6%) or non-Hispanic black (31.7%). The demographic characteristics of study participants were not significantly different from all patients admitted to the Heart Center during this period with an average age of 65.0 years (SD 15.3) and predominately male (58.6%), non-Hispanic white (62.9%) or non-Hispanic black (31.8%) The integration of the interview data with clinical data from the patient electronic medical records is currently underway. The study has received funding and ethical approval.

**Conclusions:**

Many US hospitals continue to struggle with high readmission rates in patients with cardiovascular disease. The primary objective of this study is to collect and integrate a comprehensive array of patient attributes to develop a powerful yet parsimonious model to stratify risks of rehospitalization in cardiovascular patients. The results of this research also have the potential to identify actionable targets for tailored interventions to improve patient outcomes.

## Introduction

High rates of potentially preventable hospitalizations in adults with cardiovascular disease have put enormous strain on the US health care system [1-4]. Hospital administrators and health care providers are now facing increasing pressure to develop prognostic tools to better identify patients at risk of being readmitted after discharge [2,3,5-10]. This study is an interdisciplinary project that will integrate a comprehensive set of socioeconomic, psychosocial, and behavioral factors with clinical factors to develop an effective model to stratify cardiovascular patients at risk of 30-day readmission. The specific aims of the study are twofold: to administer a novel patient survey to collect an array of nonclinical factors that will be combined with clinical factors from patient electronic medical records and to develop risk stratification models to identify key clinical and nonclinical factors associated with 30-day hospital readmissions.

## Methods

### Background and Significance

Cardiovascular disease (CVD) is the leading cause of hospitalization in adults ages 65 years and older, and readmissions after discharge are common, costly, and often preventable [1-4]. According to the American Heart Association, CVD-related illnesses cost the United States an estimated $204 billion in hospital services and physician fees in 2010 [1]. Recent studies suggest that approximately 25% of older adults with heart failure and nearly 20% of older adults with myocardial infarction (MI) are rehospitalized within 30 days of discharge, and upwards of 40% of patients are readmitted within 6 months [3-6]. Considering the enormous human and financial costs of readmissions, clinical investigators and hospital administrators are facing increasing pressure to develop prognostic tools to identify patients at risk of potentially preventable rehospitalization [11-13]. However, current strategies to predict readmissions have been largely ineffective, and the predictive prospects of many clinical variables have been nearly exhausted [3,7-10,14].

There is now considerable interest in the role of nonclinical factors in predicting early readmission [3,15-19]. However, efforts to identify novel predictors of rehospitalization have been largely unsuccessful, and hospitals continue to struggle with high readmission rates [2,5]. Of the nearly 200 studies on hospital readmissions in cardiac populations, only a handful have considered nonclinical patient attributes and their wider contexts [3,20,21]. The few studies that exist suggest that factors such as income, marital status, depressive symptoms, and living arrangement are significantly associated with readmission or death in patients with heart failure [8,14-16,22,23]. Despite these promising findings, the full scope of nonclinical variables remains relatively undefined and poorly studied. Consequently, almost nothing is known about how patients’ social resources, relationships, and behaviors outside of the hospital impact recurrent hospitalizations.

We propose to use a patient survey to identify the nonclinical characteristics of patients at Duke Heart Center. To our knowledge, this will be the first effort to integrate an array of socioeconomic, psychosocial, behavioral, and clinical factors to identify CVD patients at risk of rehospitalization. This project will lay the groundwork to help develop an effective tool to stratify cardiac patients at risk of rehospitalization prior to discharge and ultimately lower readmissions by identifying key patient characteristics that are associated with poor outcomes and markers for aggressive intervention.

### Ethics Approval

The study was approved by the Institutional Review Board (IRB) at Duke University Medical Center (protocol ID Pro00051237) and is funded by the Social Science Research Institute at Duke University.

### Design and Procedures

The study includes patients admitted for the treatment of cardiovascular-related conditions at Duke Heart Center in the Duke University Medical Center. Over the past 2 decades, Duke’s Heart Center has consistently ranked among the leading heart centers in the country (#1 in North Carolina) and is staffed by the nation’s top cardiovascular specialists who care for more than 65,000 patients each year. As a top-ranked hospital for cardiovascular care and treatment, the Heart Center’s catchment area of patients is large and diverse. Details of existing policies, initiatives, and usual-care practices at Duke University hospitals and the Heart Center (including Duke’s cardiac rehabilitation program) are extensive and fully documented elsewhere [24-27]. Duke also maintains and monitors quality scores on numerous indicators for cardiovascular outcomes, health care quality, and patient satisfaction. Cardiovascular care at Duke consistently meets or exceeds national and state averages in a number of areas, including hospital readmissions [28-30]. Additional information about ongoing cardiovascular studies at Duke University and Duke Heart Center can be found elsewhere [24,31,32].

This study will use a standardized survey to ascertain data on a comprehensive array of patient characteristics prior to discharge (see Multimedia Appendix 1). The instrument was developed to capture 5 patient-level domains: (1) patient demographics and background, (2) socioeconomic status and resources, (3) psychosocial resources, (4) health behaviors, and (5) physical and psychological status (Textbox 1).

**Measures ascertained from the patient survey.**

Demographic and socioeconomic background:

- Age (date of birth)
- Sex
- Foreign-born status
- Race/ethnicity
- Marital status
- Household size
- Educational attainment
- Employment status
- Health insurance

Psychosocial factors:

- Health literacy
- Self-efficacy toward health
- Positive outlook
- Social support
- Life stressors
- Negative outlook

Behavioral factors:

- Smoking
- Alcohol consumption
- Religious attendance
- Adherence to medications
- Place of care
- Number of hospitalizations

Self-reported health status:

- Self-rated health
- Activities of daily living limitations
- Symptoms according to the Center for Epidemiologic Studies—Depression (CES-D) survey
- Body mass index
- Likelihood of readmission
- Longevity of parents

The survey data will be collected using a brief (5-10 minute) self-administered paper questionnaire. In almost all instances, the questionnaire items were obtained from existing sources and were previously validated and shown to be psychometrically sound [33-43]. The completed surveys will be collected and the information will be entered into a standardized data entry program. The resulting database will then be linked to the patient electronic medical records using MaestroCare/DEDUCE (Duke University) to identify patients’ clinical characteristics, hospital readmissions, and mortality (when available).

Data collection will continue until the required sample size for analysis is obtained. There will be no additional contact with patients after the administration of the in-patient survey. Follow-up data collection will be limited to using MaestroCare/DEDUCE to identify the dates of hospital readmissions or mortality. Follow-up information on ambulatory or primary care will not be collected as part of the current study’s protocol, which is to identify key patient characteristics at the time of hospitalization that can be used for risk stratification prior to discharge.

The study will include a research assistant (RA) from the Division of Community Health (DCH). The RA has the appropriate background and qualifications to assist with screening for patient eligibility, obtaining informed consent, administering the survey, and data entry. As required by DCH and the Duke University Health System (DUHS), the RA will adhere to the policies and codes of conduct for DUHS employees and will have completed the required IRB Collaborative Institutional Training Initiative training (eg, informed consent), Duke Human Resources policy training and background check, and confidentiality agreements. Prior to work in the hospital, the RA also will be required to get vaccinated for influenza.

### Selection of Subjects

Consistent with Duke’s Quality Improvement initiative to identify the best practices for care, we plan to enroll all eligible subjects who are admitted to Duke University Medical Center for the treatment of cardiovascular-related illnesses (*International Statistical Classification of Diseases, 9th Edition* diagnostic codes: 390-459). Eligible subjects will be aged 18 years or older upon admission. Assuming approximately 200 patient discharges per month and a response rate comparable to in-hospital patient satisfaction surveys (≥70%) [15], we expect to enroll approximately 850 subjects during the data collection period. Reliable estimates of power/sample size are difficult to calculate for this study because of (1) the absence of true treatment groups, (2) the large number of potential predictors, and (3) unknown assumptions about the probabilities (and standard deviations) of readmission or death for each of the various covariates. Nonetheless, established methods and literature demonstrate that a sample size of approximately 500 observations and event rates of 10% to 20% should be adequate to obtain robust estimates of readmission [16,17].

### Subject Recruitment and Compensation

The study RA will screen for eligibility using the patients’ existing medical records (eg, date of birth). The study and RA will be introduced to patients by their health care provider. If patients are interested in participating, the RA will describe the study and its objectives, obtain informed consent, introduce the survey questionnaire, distribute the instrument, and collect the completed surveys. If requested, the RA will allow the subject to review the survey prior to their consent. The RA will be available to respond to patient questions and concerns throughout the consent process and the administration of the survey. Subjects who refuse to participate will be asked for the reason they declined and the RA will record any additional information (eg, age, gender) that may help minimize future refusal rates. No compensation will be provided to subjects.

### Consent Process

Consent will be obtained using standardized procedures and a signed consent form (Multimedia Appendix B). Eligible patients will be given the consent form, which can be read to the patient by the study RA and explained as needed. The designated study RA will be available to answer any questions or concerns that may arise related to the consent process or the interview itself. All subjects to be interviewed will be able to give legal consent.

### Study Interventions and Risk/Benefit Assessment

The research poses little risk to subjects and requires no interventions or invasive physical procedures. Although there is a small potential risk from loss of confidentiality, the risk of such loss will be minimized. Potential benefits of the study include the knowledge to improve patient outcomes and improve the overall quality of care at Duke Heart Center. Although there are no benefits to subjects, the results of the data collection and analysis will have potentially important implications for current medical practice and developing patient-centered approaches to treatment. Identification of the sociodemographic and behavioral characteristics of patients will be extremely useful for tailoring treatment regimens that go beyond clinical care and therapeutics by explicitly considering patients’ social resources, relationships, and environment. The implementation of a viable and effective instrument to quantify nonclinical risks based on the patients’ background has enormous potential to improve care and reduce costs associated with transitions of care and recurrent hospitalizations.

### Data Analysis and Statistical Considerations

The data will be collected and analyzed only for purposes of scientific research. As such, the data will only be used to generate statistical summaries and aggregated information that do not permit the identification of any individual patient, family, or household, either directly or inferentially. The initial stage of analysis examines the univariate and bivariate distributions of patients to characterize their baseline socioeconomic, psychosocial, behavioral, and functional status prior to discharge using *t* tests (continuous), Mann-Whitney *U* tests (nonnormal continuous), chi-square tests (categorical), and Fisher’s exact tests (binary).

The second stage of analysis will examine the factors associated with 30-day all-cause readmissions. The analyses will be conducted in several steps. First, nonparametric Kaplan-Meier plots will be used to examine the associations between the covariates and early readmission (and death). Next, competing-risk hazard models will be used to estimate the unadjusted and adjusted associations between the patient-level factors and 30-day readmissions (accounting for death as a competing risk). The final set of analyses will examine the performance of the risk models based on 2 components of accuracy. First, model calibration will determine how closely the predicted outcome agrees with the observed (actual) outcome. Graphical comparisons will be made and evaluated using Hosmer-Lemeshow chi-square goodness-of-fit tests. Next, model discrimination will be tested using Harrell’s c-index to determine the ability of the model to distinguish patients who were readmitted and those who were not [44]. At each step of the multivariate analyses, we will test for interactions among covariates to identify important subgroup variations in risks of rehospitalization. All analyses will be performed using Stata 12.0 (StataCorp LLC).

### Data and Safety Monitoring

The study only involves patient interviews and analyses with survey data and existing medical records (via MaestroCare/DEDUCE). Therefore, there are limited patient safety concerns.

### Privacy, Data Storage, and Confidentiality

The study data will be kept secure and confidential as required by law. As part of these safeguards, the data will be located and analyzed within DCH in the Department of Community and Family Medicine. To protect against the risk of loss of confidentiality, the research team will closely follow the procedures approved by the Duke University Medical Center IRB, and the data will be secured in accordance with the privacy and security regulations of the Health Insurance Portability and Accountability Act. The computerized files used for data entry and analysis will be stored on password-protected computers (and networks) in a secure office in the Mutual Building of the DCH. The DCH’s computer network is carefully protected by an appropriate firewall and a centralized monitoring system that protect access to study data. Only the principal investigator and RAs will have access to the data. The completed surveys (hard copies) and informed consent will be kept confidential and stored in locked file drawers in the Mutual Building. No participant identifiers will be used in the presentation or reporting of data.

## Results

The study was launched in February 2014 and is actively recruiting patients from the Heart Center. Approximately 550 patients have been enrolled to date and the study is expected to continue recruitment until February 2018. Preliminary analyses of study participants were conducted to compare patients currently enrolled in the study with all patients admitted during the study period and describe the preliminary distributions of study participants across key survey measures. Data collection remains ongoing, and the preliminary results presented here are provided for informational purposes for this active study protocol. Table 1 presents comparisons of hospitalized patients enrolled in the study with all eligible patients at Duke Heart Center.

Overall, results show that the 2 patient groups had similar demographic and clinical profiles. Patients enrolled in the study had a median age of 65 years (interquartile range [IQR] 19) and were predominantly male (318/520, 61.2%), non-Hispanic white (336/520, 64.6%), and married (276/520, 53.1%). The major diagnoses of diseases in patients included acute MI (58/520, 11.4%), atrial fibrillation (154/520, 30.3%), heart failure (173/520, 34.0%), hypertension (255/520, 50.1%), and diabetes (143/520, 28.1%). The demographic and disease profiles of patients were not significantly different between eligible and enrolled subjects. However, the initial patients enrolled in the study had a slightly longer median hospital stay than all patients admitted during the study period (5.1 vs 4.0 days, respectively; *P*<.001). The overall distributions of the patient characteristics ascertained from the survey are presented in Table 2.

**Table 1:** Comparison of enrolled patients with all patients admitted during the study period at Duke Heart Center (distributions were ascertained from patient electronic medical records and include all encounters (n=6880) from the 5387 total patients admitted during this period).

Parameters			All patients (n=5387)	Enrolled patients (n=520)	*P* value
**Demographic characteristics**					
	Age, median (IQR^a^)		66 (21)	65 (19)	.098
	Male, n (%)		4032 (58.60)	318 (61.2)	.255
	White, n (%)		4296 (62.85)	336 (64.6)	.422
	Married, n (%)		3722 (54.10)	276 (53.1)	.652
**Clinical characteristics**					
	**Cardiovascular diagnoses**				
		Acute MI^b^, n (%)	992 (14.42)	58 (11.4)	.059
		Atrial fibrillation, n (%)	1949 (28.33)	154 (30.3)	.353
		Heart failure, n (%)	2059 (29.93)	173 (34.0)	.054
	**Comorbid diagnoses**				
		Hypertension, n (%)	3489 (50.71)	255 (50.1)	.789
		Diabetes, n (%)	2049 (29.78)	143 (28.1)	.421
		Length of stay, median, n (%)	4.02 (4.34)	5.11 (6.9)	<.001

^a^IQR: interquartile range.

^b^MI: myocardial infarction.

**Table 2:** Characteristics of study participants admitted at Duke Heart Center (n=520).

Parameter			Values	Missing
**Demographic characteristics**				
	Age, median (IQR^a^)		66 (19)	
	Male, n (%)		318 (61.2)	
	White, n (%)		336 (64.6)	
	Married, n (%)		276 (53.1)	
	Lives alone, n (%)		139 (27.2)	8 (1.5)
**Socioeconomic characteristics**				
	High school or less education, n (%)		198 (38.5)	5 (1.0)
	**Employment status**			6 (1.2)
		Currently employed, n (%)	104 (20.2)	
		Not employed, n (%)	138 (26.9)	
		Retired, n (%)	272 (52.9)	
	**Health insurance**			7 (1.4)
		No health insurance, n (%)	10 (2.0)	
		Medicaid only, n (%)	27 (5.3)	
		Medicare, n (%)	333 (64.9)	
		Other sources, n (%)	143 (27.9)	
**Psychosocial characteristics**				
	Health literacy (0-3), mean (SD)		2.26 (0.7)	3 (0.6)
	Health self-efficacy (0-4), mean (SD)		3.23 (0.7)	3 (0.6)
	Social support (0-20), mean (SD)		16.55 (4.0)	9 (1.7)
	Life stressors (0-12), mean (SD)		3.07 (2.1)	13 (2.5)
	CES-D^b^ symptoms (0-24), mean (SD)		7.60 (4.5)	16 (3.1)
**Behavioral characteristics**				
	**Smoking history**			10 (1.9)
		Never smoked, mean (SD)	208 (40.8)	
		Past smoker, mean (SD)	249 (48.8)	
		Current smoker, mean (SD)	53 (10.4)	
	**Alcohol consumption**			5 (1.0)
		Never drinks, mean (SD)	316 (61.4)	
		Moderate consumption, mean (SD)	192 (37.3)	
		Heavy consumption, mean (SD)	7 (1.4)	
	Non-adherence to medication, mean (SD)		105 (20.9)	18 (3.5)
**Health-related characteristics**				
	BMI^c^, mean (SD)		30.33 (8.0)	2 (0.4)
	ADL^d^ disability, mean (SD)		290 (57.4)	15 (2.9)
	Diagnosed HTN^e^, mean (SD)		255 (49.0)	
	Diagnosed diabetes, mean (SD)		143 (27.5)	
Readmission at 30 days, mean (SD)			105 (20.2)	

^a^IQR: interquartile range.

^b^CES-D: Center for Epidemiologic Studies—Depression scale.

^c^BMI: body mass index.

^d^ADL: activities of daily living.

^e^HTN: hypertension.

Results show that large percentages of admitted patients were not married (244/520, 46.9%), lived alone (139/520, 27.2%), had a high school education or less (198/520, 38.5%), and were not employed (138/520, 26.9%). Although the majority of patients were Medicare beneficiaries (333/520, 64.9%), some had no health insurance (10/520, 2.0%) or only Medicaid coverage (27/520, 5.3%). Most patients had a history of smoking, with nearly half who quit smoking (249/520, 48.8%) and 10.4% (53/520) who currently smoke. Most patients reported no alcohol consumption (316/520, 61.4%) and very few reported heavy consumption (7/520, 1.4%). More than 1 in 5 patients (105/520, 20.9%) reported not taking their prescribed medication in the past year.

In terms of health status, patients had an average body mass index of 30.3 and a sizeable percentage of patients had some limitation in activities of daily living (290/520, 57.4%), were diagnosed with hypertension (255/520, 49.0%), or were diagnosed with diabetes mellitus (143/520, 27.5%). Preliminary results also show that approximately 20.2% (105/520) of patients currently enrolled in the study were readmitted within 30 days of discharge. The current readmission rate of study participants is consistent with national estimates for cardiovascular patients and with readmission rates documented at other North Carolina hospitals [29,30].

Overall, preliminary results show that missing data were minimal (≤3%) across measures in the patient survey. Patient enrollment and data collection efforts remain ongoing. Further integration and analysis of the patient clinical data and survey interview data are also currently underway.

## Discussion

### Summary

Cardiovascular disease is the leading cause of (re)hospitalization in older adults and despite enormous investments and a plethora of research, many US hospitals continue to struggle with high readmission rates [1,2]. The purpose of this paper was to present the protocol for the implementation of a study to identify how a wide array of socioeconomic, psychosocial, behavioral, and clinical factors are associated with risks of 30-day readmission in patients with cardiovascular disease. The results of this interdisciplinary research have the potential to identify actionable targets for tailored interventions to improve patient outcomes.

In 2009, the American College of Cardiology and the Institute for Healthcare Improvement launched the Hospital to Home (H2H) national campaign to reduce readmissions and improve the transitioning of care for individuals hospitalized with CVD [45]. The overarching goal of the H2H initiative is to reduce rehospitalizations in cardiovascular patients by 20% in the coming years. Failure to meet this challenge may result in the loss of Medicare reimbursement for these untimely readmissions. According to recent estimates, more than one-fifth of older adults with heart failure and acute MI are readmitted within 30 days of discharge and almost 40% are rehospitalized within 6 months [3,5,6,45,46]. Mortality rates after discharge are similarly high [5,9,45]. Although studies show that early physician follow-up, counseling, and improved discharge planning can improve patient outcomes and lower subsequent readmissions [4,47,48], these efforts are often unsustainable due to the prohibitive costs of broad interventions. Thus, it has become increasingly necessary to target patients who are at greatest risk of negative outcomes.

Results from this interdisciplinary study have the potential to assist in clinical decision making, improve transitions of care, reduce hospital costs (and reimbursement penalties), and improve the lives of those with CVD. The proposed research will develop an integrated model that can identify the profiles of patients at greatest risk of rehospitalization or death after discharge. Although not all socioeconomic, psychosocial, and behavioral factors are amenable to medical intervention, identifying the key factors—and how they constellate—will provide actionable knowledge that can be used to devise effective approaches to treatment and rehabilitation (Table 3).

For example, patients with low education may benefit from health-literacy programs to improve their ability to understand complex treatment strategies and manage disease. Alternatively, patients who are socially isolated may benefit most from group therapy to enhance rehabilitative efforts and provide social support. It also may be that psychological distress is an underlying cause of excess alcohol, tobacco, or food intake, and efforts to reduce or manage stress may present the widest prognostic value.

This study will help lay the scientific groundwork for implementing a risk assessment tool that will have important implications for medical practice and improving patient outcomes. The goal of this project is to identify key nonclinical risk factors that can then be integrated with known clinical risk factors from patient medical records to produce a fast and accurate method of risk classification prior to hospital discharge. For physicians, a robust prognostic tool will allow them to quickly identify and aggressively treat high-risk patients who may have otherwise gone undetected through standard processes of care. For hospitals, improved patient stratification and targeted care will help lower the significant costs of emergency room visits and rehospitalizations in those with potentially preventable relapses. And for patients, improved risk assessment will not only facilitate the highest level of personalized care but will also provide them knowledge of health risks that go beyond the cautionary litany of poor diet, inactivity, and smoking.

**Table 3:** Examples of areas for intervention from study results.

Categories	Identified risks	Possible interventions
Socioeconomic factors	Low education	Provide educational resources and instruction (eg, coaches) to improve health literacy to better manage medications and treatment in low-educated patients
Psychosocial factors	Depression	Provide psychological counseling and schedule group meetings to improve coping strategies and social support in depressed patients
Behavioral factors	Physical inactivity	Implement aerobic exercise interventions to improve cardiorespiratory fitness in sedentary patients
Clinical factors	Hypertension	Schedule routine follow-ups and provide access to coaching/tele-coaching programs to monitor blood pressure control and medication adherence in hypertensive patients
Interactive risks	Widowed×Diabetes	Combine routine physician visits with group sessions to monitor diabetes maintenance and provide social support to minimize complications and treatment noncompliance in widowed diabetics
Cumulative risks	≥3 Behavioral risks	Implement behavioral therapy sessions that use support systems allowing patients to self-select behaviors most likely to achieve risk reduction (in number rather than type)

### Limitations

We also acknowledge limitations of this study. First, we recognize that the study is limited to patients admitted for cardiac care at Duke Heart Center; therefore, the generalizability of the findings will require further research. Second, the patient survey does not include an exhaustive list of potential factors that may be associated with rehospitalization or other poor outcomes. Rather, the survey includes a wide range of patient characteristics as an important step toward identifying and quantifying major components of patients’ nonclinical background (eg, their education, living arrangement, health literacy, social support) to develop real-time and real-world profiles of CVD patients who are most vulnerable during periods of transitional care. Additional research should build on these findings to further identify and refine such factors. Third, preliminary analyses suggest that initial patients enrolled in the study were hospitalized approximately one day longer than patients who were not enrolled in the study—possibly because of the greater opportunity for study recruitment with a longer length of stay (LOS). Analyses during study enrollment will continue to examine potential differences in LOS, and if differences persist, our subsequent readmission models will assess whether such variations have a (moderating) influence on the associations between risk factors and 30-day readmission. Finally, post hoc power analyses will be conducted near the completion of data collection to determine if the number of patients is sufficient to detect significant differences among covariates. If required, an IRB amendment will be submitted to continue patient enrollment.

### Conclusion

In sum, the objectives of this study are highly aligned with the National Institute of Health (NIH) mission of improving transitions of care and reducing hospital costs through the identification and quantification of cardiovascular risks. A major goal of NIH and Healthy People 2020 is to understand the social, psychosocial, and behavioral determinants of adverse outcomes in adults with CVD and to reduce the burden of disease in vulnerable segments of the population. A patient-centered model that can effectively identify and stratify those at risk of rehospitalization will have enormous potential to assist in clinical decision making, reduce hospital costs, and ultimately, improve the lives of those with cardiovascular illness. We are confident that the proposed research will significantly contribute to the interdisciplinary science necessary to help achieve these goals.

## Appendix

Multimedia Appendix 1 Patient survey.

Multimedia Appendix 2 Consent form.

## Abbreviations

ADL: activities of daily living
CES-D: Center for Epidemiological Studies—Depression scale
CVD: cardiovascular disease
DCH: Division of Community Health
DUHS: Duke University Health System
H2H: Hospital to Home
IRB: Institutional Review Board
IQR: interquartile range
LOS: length of stay
MI: myocardial infarction
NIH: National Institutes of Health
RA: research assistant
