# Supplementary material for: Identifying Nonclinical Factors Associated With 30-Day Readmission in Patients with Cardiovascular Disease: Protocol for an Observational Study
Source: JMIR Res Protoc. 2017 Jun 15;6(6):e118. doi: 10.2196/resprot.7434 (PMC5491895; doi:10.2196/resprot.7434)
Supplement: Multimedia Appendix 1 [file resprot_v6i6e118_app1.pdf]

Duke Heart Center

For office use

Today's date: ____/____/____
mm / dd / yy

Background

Sex
☐ Male ☐ Female

Year of birth: _______
yyyy

Current marital status
☐ Married ☐ Divorced
☐ Separated ☐ Widowed
☐ Never Married ☐ Other ____________

Race/Ethnicity (check all that apply)
☐ White/Caucasian ☐ Hispanic
☐ Black/African American ☐ Asian
☐ American Indian/Alaska Native ☐ Other _______________
☐ Native Hawaiian/Pacific Islander

Current health insurance coverage (check all that apply)
☐ Employer provided ☐ Medicare
☐ Buy insurance directly ☐ Medicaid
☐ VA or other military program ☐ Indian Health Service
☐ Other ________

In what country were you born? ☐ U.S. ☐ Other________________

1. Where do you usually go for your health care?

Primary care clinic or doctor's office	Specialist clinic	VA	Free clinic	Urgent care	Emergency room	Other
☐	☐	☐	☐	☐	☐	☐

2. Overall, how difficult is it for you to get routine medical care (in a hospital/clinic) when needed?

Extremely difficult	Moderately difficult	Somewhat difficult	Not very difficult	No problem at all
☐	☐	☐	☐	☐

a. If difficult, what is the primary reason?

Transportation	Cost	Work issues	Family issues	Other
☐	☐	☐	☐	☐

3. In the past year, how many times have you been hospitalized (overnight)?

Not hospitalized	Once	2-3 times	4-5 times	More than 5 times
☐	☐	☐	☐	☐

4. How much do you agree or disagree with the following:

	Strongly agree	Agree	Neither agree nor disagree	Disagree	Strongly disagree
Keeping healthy depends on things that I can do myself	☐	☐	☐	☐	☐
There are certain things I can do for myself to reduce the risk of a future heart attack or health problem	☐	☐	☐	☐	☐
I work hard at trying to stay healthy	☐	☐	☐	☐	☐
When I am sick, getting better is pretty much in the doctor's hands	☐	☐	☐	☐	☐

5. The following is a list of activities that people may have difficulty with because of a health or physical problem. Please look over the activities listed below and indicate how difficult each activity is for you.

	Not at all difficult	A little difficult	Somewhat difficult	Very difficult/ Cannot do		[IF NEEDED] is someone there to help you?
Walking across the room	☐	☐	☐	☐	⇨	☐ Yes ☐ No
Getting dressed	☐	☐	☐	☐	⇨	☐ Yes ☐ No
Showering or bathing	☐	☐	☐	☐	⇨	☐ Yes ☐ No
Eating	☐	☐	☐	☐	⇨	☐ Yes ☐ No
Getting in and out of bed	☐	☐	☐	☐	⇨	☐ Yes ☐ No
Toileting	☐	☐	☐	☐	⇨	☐ Yes ☐ No

6. Overall, how would you rate your health?

Excellent	Very good	Good	Fair	Poor
☐	☐	☐	☐	☐

7. The following questions are about stress in your life. Stress is defined as feeling irritable or anxious, or as having difficulty sleeping.

	Never stressed	Occasionally feel stress	Often feel stress	Always feel stress
How often do you feel stress at work?	☐	☐	☐	☐
How often do you feel stress at home?	☐	☐	☐	☐
How often do you feel stress because of financial concerns?	☐	☐	☐	☐
How often do you feel stress because of your health?	☐	☐	☐	☐

8. Are your parents still living?

a. Mother ☐ Yes ☐ No ⇨ [IF NO] at what age did she die? _____

a. Father ☐ Yes ☐ No ⇨ [IF NO] at what age did he die? _____

9. The following questions ask about people who provide you with assistance and support.

	None of the time	A little of the time	Some of the time	Most of the time	All of the time
Is there someone available to you whom you can count on to listen to you when you need to talk?	☐	☐	☐	☐	☐
Is there someone available to you to give you good advice about a problem?	☐	☐	☐	☐	☐
Is there someone available to you who shows you love and affection?	☐	☐	☐	☐	☐
Is there someone available to you to help you with daily chores?	☐	☐	☐	☐	☐
Can you count on anyone to provide you with emotional support (talking over problems or helping make a difficult decision)?	☐	☐	☐	☐	☐
Do you have as much contact as you would like with someone you feel close to, someone in whom you can trust and confide?	☐	☐	☐	☐	☐

10. How often do you attend religious services?

Not at all	One or more times a year	2 or 3 times a month	Once a week	More than once a week
☐	☐	☐	☐	☐

11. The following is a list of ways you may have felt. Please indicate how often you have felt this way during the past week.

	Rarely or none of the time	Some of the time	Most of the time	All or almost all of the time
You felt depressed	☐	☐	☐	☐
You felt that everything you did was an effort	☐	☐	☐	☐
Your sleep was restless	☐	☐	☐	☐
You were happy	☐	☐	☐	☐
You felt lonely	☐	☐	☐	☐
You enjoyed life	☐	☐	☐	☐
You felt sad	☐	☐	☐	☐
You could not get “going”	☐	☐	☐	☐

12. Do you have a pet? ☐ Yes ☐ No

13. How often do you have someone (like a family member, friend, hospital/clinic worker, or caregiver) help you read hospital materials?

All of the time	Most of the time	Some of the time	A little of the time	None of the time
☐	☐	☐	☐	☐

14. How often do you have problems learning about your medical condition because of difficulty understanding written information?

All of the time	Most of the time	Some of the time	A little of the time	None of the time
☐	☐	☐	☐	☐

15. How confident are you filling out forms by yourself?

Very confident	Confident	Not very confident	Not at all confident
☐	☐	☐	☐

16. Please indicate your agreement with the following statements

	Strongly disagree	Disagree	Neither agree nor disagree	Agree	Strongly agree
In difficult or hard times, I usually expect the best.	☐	☐	☐	☐	☐
If something can go wrong for me, it will.	☐	☐	☐	☐	☐
I’m always optimistic about my future.	☐	☐	☐	☐	☐
I hardly ever expect things to go my way.	☐	☐	☐	☐	☐
I rarely count on good things happening to me.	☐	☐	☐	☐	☐
Overall, I expect more good things to happen to me than bad.	☐	☐	☐	☐	☐

17. In the past year, how often have you not taken a medication that your doctor prescribed because of cost, side-effects, or any other reason?

Always	Frequently	Occasionally	Rarely	Never
☐	☐	☐	☐	☐

---

18. In general, how often do you have a drink containing alcohol? A "drink" can be a can or bottle of beer, glass of wine, a cocktail, or a shot of hard liquor.

Never drink	Less than 1 drink a day	1-2 drinks a day	3-4 drinks a day	5 or more drinks a day
☐	☐	☐	☐	☐

---

19. Which of the following best describes your cigarette smoking status?

Never smoked	Stopped smoking more than 1 year ago	Stopped smoking less than 1 year ago	Smoked (even a puff) in the past 30 days
☐	☐	☐	☐

[If smoked cigarettes at all in the past 30 days]

a. On average, how many cigarettes did you smoke each day? _____

---

20. What is your current height and weight?

Height: ______ Weight: ______

---

21. What is the highest level of education you have completed?

No formal schooling	Elementary (1-8 years)	Less than high school	High school graduate or GED	Some college or vocational school	College graduate	Post-graduate education
☐	☐	☐	☐	☐	☐	☐

---

22. What is your current employment status?

Working full-time	Working part-time	Unemployed	Homemaker	Retired	Disabled	Other
☐	☐	☐	☐	☐	☐	☐

---

23. How many people (including yourself) live in your household? _____ # of persons

---

24. How likely do you think you will return to the hospital for an unplanned or emergency visit within the next 30 days?

Very likely	Somewhat likely	Somewhat unlikely	Not very likely
☐	☐	☐	☐

- Thank you for your cooperation -
