# Supplementary material for: Identifying Nonclinical Factors Associated With 30-Day Readmission in Patients with Cardiovascular Disease: Protocol for an Observational Study
Source: JMIR Res Protoc. 2017 Jun 15;6(6):e118. doi: 10.2196/resprot.7434 (PMC5491895; doi:10.2196/resprot.7434)
Supplement: Multimedia Appendix 2 [file resprot_v6i6e118_app2.pdf]

DUKE UNIVERSITY HEALTH SYSTEM

Consent To Participate In A Research Study
Non-clinical Factors Associated with Rehospitalization in Patients at Duke Heart Center

You are being asked to take part in this research study because you have heart disease. Research studies are voluntary and include only people who choose to take part. Please read this consent form carefully and take your time making your decision. As your study doctor or study staff discusses this consent form with you, please ask him/her to explain any words or information that you do not clearly understand. We encourage you to talk with your family and friends before you decide to take part in this research study. The nature of the study, risks, inconveniences, discomforts, and other important information about the study are listed below.

Please tell the study doctor or study staff if you are taking part in another research study.

This research is being conducted by investigators in the Duke University Health System (DUHS) in collaboration with the Duke Heart Center, Duke Clinical Research Institute, and the Duke Division of Community Health.

WHO WILL BE MY DOCTOR ON THIS STUDY?
Dr. Matthew Dupre is the Principal Investigator (PI) on the study.

WHY IS THIS STUDY BEING DONE?
The purpose of this study is to collect information from patients to better understand the factors that may help individuals better manage their heart condition after being discharged. By participating in a brief survey, you will provide new information that will allow us to identify the non-clinical factors that may be associated with preventable rehospitalizations in patients at Duke Heart Center. Your participation is very important to us and will help us to continue delivering the best available treatment and the highest quality of care to patients at Duke.

HOW MANY PEOPLE WILL TAKE PART IN THIS STUDY?
Approximately 850 people will take part in this study at Duke Heart Center.

WHAT IS INVOLVED IN THE STUDY?
If you agree to be in this study, you will be asked to sign this consent form. As part of the study, you are being asked to participate in a 5-10 minute survey that will allow you share your background, experiences, and concerns related to your health and well-being. Your responses will be combined with information from your medical record to better understand your health and health needs. We will then group the responses from all participants to identify common characteristics that will help us determine whether some patients may benefit from additional care. The project staff is available to explain any information in this consent form or the survey that is not clear. Your participation is entirely voluntary and refusal to complete the survey will not affect your care in any way.

HOW LONG WILL I BE IN THIS STUDY?
The study will collect information from patients over the next 6 months. Participation in the study requires only the completion of a single survey and will not require follow-up information or contact. You can choose to stop participating at any time without penalty or loss of any benefits to which you are entitled. However, if you decide to stop participating in the study, we encourage you to talk to your doctor first.

WHAT ARE THE RISKS OF THE STUDY?
The research poses little to no risk to participants. There is a small potential risk from loss of confidentiality. However, the information provided on the survey will be kept secure and confidential as required by law. The

completed surveys will be stored in locked file drawers and the computerized data will only be used to generate summary information that does not permit the identification of any individual patient, family, or household, either directly or indirectly.

ARE THERE BENEFITS TO TAKING PART IN THE STUDY?
Although there are no direct benefits to study participants, the results of this study will have potentially important implications for developing patient-centered approaches to treatment and care. Taking part in this study will provide us valuable information on the characteristics of patients, like you, who may benefit from additional care to better manage their heart condition. We hope that in the future the information learned from this study will benefit other people with your condition.

WILL MY INFORMATION BE KEPT CONFIDENTIAL?
Study records that identify you will be kept confidential as required by law. Federal Privacy Regulations provide safeguards for privacy, security, and authorized access. Except when required by law, you will not be identified by name, social security number, address, telephone number, or any other direct personal identifier in study records disclosed outside of DUHS. For records disclosed outside of DUHS, you will be assigned a unique code number. The key to the code will be kept in a locked file in Dr. Matthew Dupre's office.

As part of the study, Dr. Matthew Dupre and his study team will use existing information from your medical records. This information may include diagnostic tests, labs results, and imaging tests to measure the function of your heart, liver, and kidneys. In addition, your records may be reviewed in order to meet federal or state regulations. Such reviewers may include the Duke University Health System Institutional Review Board. If any group reviews your research record, they may also need to review your entire medical record.

The study results will be retained in your research record for at least six years after the study is completed. At that time either the research information not already in your medical record will be destroyed or information identifying you will be removed from such study results at DUHS. Any research information in your medical record will be kept indefinitely.

If this information is disclosed to outside reviewers for audit purposes, it may be further disclosed by them and may not be covered by the federal privacy regulations.

While the information and data resulting from this study may be presented at scientific meetings or published in a scientific journal, your identity will not be revealed.

WHAT ARE THE COSTS?
There are no costs for participation in this study.

WHAT ABOUT COMPENSATION?
No compensation will be provided for participation in this study.

DUKE UNIVERSITY HEALTH SYSTEM

Consent To Participate In A Research Study
Non-clinical Factors Associated with Rehospitalization in Patients at Duke Heart Center

WHAT ABOUT MY RIGHTS TO DECLINE PARTICIPATION OR WITHDRAW FROM THE STUDY?
You may choose not to be in the study, or, if you agree to be in the study, you may withdraw from the study at any time. If you withdraw from the study, no new data about you will be collected for study purposes other than data needed to keep track of your withdrawal.

Your decision not to participate or to withdraw from the study will not involve any penalty or loss of benefits to which you are entitled, and will not affect your access to health care at Duke. If you do decide to withdraw, we ask that you contact Dr. Matthew Dupre in writing and let him know that you are withdrawing from the study. His mailing address is: 411 W. Chapel Hill Street, DUMC Box 104425, Durham, NC 27701.

We will tell you about new information that may affect your health, welfare, or willingness to stay in this study.

WHOM DO I CALL IF I HAVE QUESTIONS OR PROBLEMS?
For questions about the study or a research-related injury, or if you have problems, concerns, questions or suggestions about the research, contact Dr. Matthew Dupre and ask to have him paged at 919-681-6811 during regular business hours and at 919-480-7051 after hours and on weekends and holidays.

For questions about your rights as a research participant, or to discuss problems, concerns or suggestions related to the research, or to obtain information or offer input about the research, contact the Duke University Health System Institutional Review Board (IRB) Office at (919) 668-5111.

STATEMENT OF CONSENT
"The purpose of this study, procedures to be followed, risks and benefits have been explained to me. I have been allowed to ask questions, and my questions have been answered to my satisfaction. I have been told whom to contact if I have questions, to discuss problems, concerns, or suggestions related to the research, or to obtain information or offer input about the research. I have read this consent form and agree to be in this study, with the understanding that I may withdraw at any time. I have been told that I will be given a signed and dated copy of this consent form."

____ __________ __________
Signature of Subject Date Time

____ __________ __________
Signature of Person Obtaining Consent Date Time
